# Special Issue: Vaccines and Animal Health

**DOI:** 10.3390/vaccines14010041

**Published:** 2025-12-30

**Authors:** Matteo Legnardi, Claudia Maria Tucciarone

**Affiliations:** Department of Animal Medicine, Production and Health (MAPS), University of Padua, Viale dell’Università 16, 35020 Legnaro, Italy

Veterinary medicine operates at the frontline of a constantly evolving biological landscape, where pathogens—particularly viruses—display remarkable genetic plasticity and adaptive capacity, which enable them to evade host immunity, cross species barriers, and establish persistent or recurring infections.

Livestock sectors, in particular, represent environments where high animal density, genetic homogeneity, and productive chain permeability create optimal conditions for pathogen amplification and circulation, often spreading rapidly within and between farms. In this context, traditional biosecurity measures, while essential, can be insufficient on their own to fully contain infections or prevent their spread. Therefore, vaccination represents an invaluable tool in veterinary medicine—not only as a mean of protecting animal health and welfare, but also as a cornerstone of disease control strategies aimed at reducing pathogen circulation, which inherently provides opportunities for pathogen evolution, reassortment, and host adaptation, potentially leading to the emergence of novel variants.

In this context, the comprehensive review by Wang and Feng (2024) [1] effectively illustrated the long and complex path undertaken by veterinary medicine in addressing the issue of Porcine Reproductive and Respiratory Syndrome (PRRS), a widespread and impactful disease. By systematically presenting the stages of research and results aimed at achieving control of this clinical and production challenge, the authors underline the multifactorial nature of PRRS, highlighting the importance of vaccination for its control.

Aside from being pivotal at protecting animals against diseases, vaccines may also serve as a precious resource to reduce infection pressure, thus limiting the ecological and evolutionary space in which zoonotic pathogens may emerge or persist. A good example is the production of a chimeric protein-based vaccine candidate against Shiga toxin-producing *Escherichia coli* (STEC) by Vidal et al. (2024) [2] that aims to protect piglets against a primary cause of disease by enhancing lactogenic immunity through sow immunization. The reduction in piglet susceptibility has positive implications on the pathogen maintenance in the swine reservoir, thus contributing to containing the zoonotic risk.

The human–animal interface does not involve only the productive compartment, and at the domestic level, the close proximity between pets and humans—often sharing the same environment and lifestyle—creates additional pathways for pathogen exchange. On the other hand, the existence of shared microbiological challenges and disease patterns across animal and human pathogens can set the conditions for the identification of common perspectives in terms of prevention and control strategies, possibly providing models to evaluate vaccine platforms and innovations. In this context, Brostoff et al. (2024) [3] offered preliminary data about an mRNA vaccine for feline coronavirus (FCoV) immunization, using an uncommon platform in veterinary medicine to protect against a challenging disease, for which no safe and really effective vaccine has been produced yet. Another RNA-based platform, an RNA Particle vaccine, was evaluated to immunize dogs against canine influenza virus (CIV) by Classe et al. (2024) [4], proposing a safe, low dose, strongly immunizing and non-adjuvanted vaccine, as an alternative to traditional inactivated ones.

Regarding innovations, vaccine administration route is also a developing research field, both in human and veterinary medicine. Lee et al. (2024) [5] have compared the immune response to foot-and-mouth disease virus (FMDV) vaccines administered by intramuscular (IM) and intradermal (ID) routes, evidencing the lack of lesions and a similar protection level even though a much lower dose was used via ID, leading to reduced costs. To encompass production limits, Berryman et al. (2025) [6] have addressed the time-consuming problem of FMDV adaptation to baby hamster kidney cells (BHK) by generating chimeric viruses based on field-strains inserting specific amino acid substitutions for cell culture adaptation. This study offers preliminary indications on a research and development pathway that could allow a more rapid response to outbreaks of a severe and impactful disease. On the other hand, Kim et al. (2024) [7] expanded on the features of current vaccines against FMD as well as lumpy skin disease (LSD), whose adoption may be hampered by fear of adverse reactions. The analysis of acute phase responses highlighted the need for a longer follow-up to monitor adverse reactions elicited by LSD vaccines, improving our understanding and possibly helping reduce vaccine hesitancy.

Aside from infectious diseases, vaccination is essential for the control of many parasitic diseases, deserving similar research efforts to improve available solutions. In this context, Mi et al. (2024) [8] investigated the potential of Elongation factor 2 (EF-2) as a candidate antigen for the development of multivalent vaccines targeting mixed infections by *Eimeria* species; Hasan et al. (2023) [9] tested a nanoparticle-based vaccine against haemonchosis in a murine model; and Li et al. (2024) [10] applied calcium mineralization on *Toxoplasma gondii* tachyzoites to improve their stability and reduce toxicity. In all three studies, the obtained results offer promising glimpses on future vaccination approaches.

Lastly, Birkmann et al. (2024) [11] explored an unconventional application of vaccines to fight non-transmissible diseases. In particular, they observed how virus-like particle-based vaccines targeting interleukin-5 (IL-5), developed to combat allergic conditions in horses, may also be effective in preventing and cure chronic urticaria, shedding light on the unexpected role of eosinophils in this reaction.

Insights gained from veterinary vaccination programs—ranging from mass immunization strategies in livestock to targeted vaccination in companion animals—have historically informed and continue to inform translational approaches applicable to human medicine. In this sense, veterinary vaccinology represents a critical bridge between basic immunological research and public health implementation, reinforcing the One Health paradigm and highlighting the reciprocal benefits of cross-sectoral knowledge exchange.
